# Use of transpyloric feeds in extremely low birth weight infants at risk of severe bronchopulmonary dysplasia—a single center experience

**DOI:** 10.3389/fped.2024.1496620

**Published:** 2024-11-29

**Authors:** Ahana Nagarkatti, Shikha Sarkar, Amirul Anuar, Naveed Hussain

**Affiliations:** ^1^ College of the Holy Cross, Worcester, MA, United States; ^2^ Neonatology, Connecticut Children's Medical Center, Hartford, CT, United States; ^3^ Biostatistics, Connecticut Children's Medical Center, Hartford, CT, United States

**Keywords:** ELBW (exterme low birth weight infants), transpyloric feeding, sBPD, LPHN, Gastrostomy Tubes, Tracheostomy Tubes

## Abstract

**Introduction:**

The incidence of severe BPD (sBPD), defined as needing oxygen or positive pressure at 36 weeks corrected gestational age (CGA), has remained unchanged. These infants are at risk for developing late pulmonary hypertension (LPHN) or needing surgical interventions such as Gastrostomy Tubes (GT) or Tracheostomy Tubes (TT). The finding of pepsin in the lungs of infants who were extremely low birth weight (ELBW) with sBPD has led to the speculation that gastroesophageal reflux (GER) and aspiration could contribute to their lung disease. Micro-aspiration-reducing strategies such as Transpyloric feeds (TpF) have not been well studied.

**Objectives:**

To compare ELBW infants with sBPD managed with or without TpF and determine the difference between the two groups for (i) illness severity, (ii) LPHN, (iii) need for GT, and (iv) TT; the secondary aim was to study the TpF group to (i) evaluate the change in Respiratory Severity Score (RSS) before and after TpF, and (ii) evaluate the time taken to affect the change in RSS.

**Methods:**

In this retrospective study there were 229 ELBW infants with sBPD (78 in the TpF group, 151 in the non-TpF group). SPSS software was used for univariate analyses.

**Results:**

There was no difference in sex or race. TpF group had (i) a lower BW, GA, higher severity of illness (ii) higher incidence of LPHN (*p* < 0.05), (iii) higher need for GT (*p* < 0.001) and TT (*p* < 0.001). In the TpF group, 60 who were on TpF for pulmonary protection from micro aspiration (lung protection group), had significantly improved RSS (*p* < 0.05), and symptoms within 45 days in 57 out of 60 infants (95%). They improved their respiratory status by 14 days, and 80% of responders could be identified by 21 days after initiation. In the 18 that TpF was started for documented airway protection (airway protection group), there was a higher need for GT or TT.

**Conclusions:**

TpF could play an essential role in the management of ELBW infants with sBPD. Considering the limitations of a single center retrospective study, prospective randomized control trials are needed to confirm these findings.

## Introduction

Bronchopulmonary dysplasia (BPD) is a significant complication of prematurity, characterized by abnormal lung development and function, often requiring prolonged respiratory support and higher caloric support (1). The incidence of BPD has remained relatively stable over the past decades despite advances in neonatal care (2). Severe BPD (sBPD), defined as the need for continuing respiratory support beyond 36 weeks corrected gestational age (CGA), accounts for a substantial burden on healthcare resources due to the need for frequent rehospitalizations and persistent respiratory symptoms even into adolescence (2). Strategies to prevent or manage sBPD have included various feeding and nutritional interventions, including high calorie and protein intake and transpyloric feeds (TpF) (1, 3).

Micro aspiration of gastric contents has been implicated in the evolution of sBPD (4). Bypassing the stomach with TpF has been used in adults to minimize the effect of refluxed gastric contents (5, 6). TpF has been used in Pediatric and Adult intensive care units for critically ill patients. The method of infusion of enteral feeding (gastric or transpyloric) is based on institutional factors (related to protocols and available expertise) and the degree of risk and potential tolerance of the individual patient (6). Continuous TpF during weaning from the ventilator and tracheal extubation is safe and effective in delivering optimal nutrition (7, 8). Similar to findings in older children and adults, TpF has also been shown to be a safe and effective means of early nutritional intake in low-birth-weight infants (9).

The timing of initiating TpF in infants who are extremely low birth weight (ELBW) appears to be important in influencing the outcomes of interest. Early TpF initiation has focused on preventing sBPD. At the same time, late initiation around 36 weeks CGA has aimed to mitigate the development of Late Pulmonary Hypertension (LPHN) and decrease the need for Tracheostomy Tubes (TT) and Gastrostomy Tubes (GT) (9–12). Additionally, some studies have used TpF to manage patients needing airway protection from aspiration due to demonstrable uncoordinated oral feeds or due to gastroesophageal reflux (GER) (6, 13–15). Early TpF initiated within the first week after birth has shown a reduced risk of death or BPD, fewer days of mechanical ventilation, fewer umbilical line days, and less prolonged use of antibiotics than gastric feeding (9).

TpF has also been tried in the management of infants with BPD with mixed results (16). Studies have shown that TpF may help with apnea related to GER, improve weight gain and reduce oxygen requirements in preterm infants with BPD (9, 17, 18). Other studies have found that TpF may be detrimental because of increased hypoxemia and worse outcomes at discharge (10, 19, 20). Additionally, there is concern that TpF may be associated with an increased risk of necrotizing enterocolitis (NEC), feeding intolerance, and gastrointestinal bleeding (13). Lack of details, especially on tube type, patient selection, and timing of initiation, preclude a rigorous evaluation of these studies (21). Therefore, more research is needed to determine the effectiveness and safety of TpF in the management of sBPD.

The use of guideline-based initiation of TpF at our center provided an opportunity to retrospectively evaluate the efficacy and safety of TpF in a pre-selected population of NICU patients. At our institution, the guidelines instituted for ELBW infants approaching 36 weeks' CGA included: (i) monitoring of cardiac effects of evolving lung disease with serum proBNP and cardiac echocardiography, (ii) considering the use of diuretics and/or post-natal steroids for improving lung compliance, and (iii) considering initiation of TpF to mitigate effects of potential micro aspiration related lung injury.

The present study was designed with two main aims. First, to compare ELBW infants with sBPD managed with or without TpF and determine the difference between the two groups for (i) illness severity, (ii) LPHN, (iii) need for GT, and (iv) TT. The secondary aim was to study the TpF group to (i) evaluate the change in Respiratory Severity Score (RSS) before and after TpF, and (ii) evaluate the time taken to affect the change in RSS.

## Material and methods

### Study site and duration

This retrospective study was based on admissions and transfers of ELBW infants from regional hospitals to the Connecticut Children's Medical Center's (CCMC) Neonatal Intensive Care Unit (NICU) in Hartford, a level 4 referral center, between January 2010 and June 2023.

### Eligibility

All ELBW survivors at 36-week CGA with evolving BPD were included. Our definition of sBPD was the need for continuing respiratory support beyond 36 weeks CGA. We excluded the following: (i) infants who died or were transferred out of the NICU before 36 weeks CGA, (ii) ELBW infants without evolving BPD at 36 weeks CGA, and (iii) infants with pre-existing gastrointestinal problems, including spontaneous intestinal perforation, NEC, major gastrointestinal surgery and short gut syndrome.

### TpF protocol

TpF was initiated based on the medical team's judgment or the above-mentioned institutional guidelines for ELBW infants around 36 weeks CGA. All TpF tubes were Avanos Neomed Feeding Tubes® made of polyurethane in sizes 5, 6.5, and 8 (non-weighted) that were placed by the bedside nurse and confirmed by radiographs. TpF tubes that were accidentally dislodged were replaced, and their position was reconfirmed. The decision to remove the TpF tube was based on significant clinical improvement, GT surgery, or elective removal after 4–6 weeks without improvement as per the institutional guidelines.

### Data collection

Daily clinical data during this period was available from electronic charts (Neonatal Information System-5, Medical Data Systems, Philadelphia, PA) that were updated daily and checked for accuracy at the time of the infant's discharge by a medical data team of neonatal nurses. Data definitions were based on the Vermont-Oxford Network (VON) Manual of Operations. [2022_Manual_of_Operations__Part_2__Release_26.2_PDF.pdf (zendesk.com)] The definitions for the variables used in this study did not materially change during the study period. Data not available on the NIS-5 database were obtained from a manual chart review on EPIC® patient records. The study was approved by the Connecticut Children's Institutional Review Board.

Demographic, morbidity, respiratory support, and medication data were collected, and the Respiratory Severity Score (RSS) was calculated before the initiation of TpF and at subsequent times during the hospital stay. RSS, a previously validated measure of respiratory severity, was calculated as a product of Mean Airway Pressure (MAP) and Fraction of Inspired Oxygen (FiO_2_) (22). Details collected relating to TpF included duration, type of feeds, feed intolerance, NEC, perforation, and other gastrointestinal complications. Outcomes before hospital discharge were evaluated, especially for the development of LPHN and the need for a surgically placed GT or TT.

After ascertaining the safe osmotic load of feeds, it was determined that there was no need for decreasing caloric density of feeds during TpF. Detailed evaluation of nutrition and growth was beyond the scope of this study.

### Data analysis

SPSS (Version 29, IBM Corp., Poughkeepsie, NY) software was used for statistical analyses. Group comparisons were made with univariate analyses (*χ*^2^ Tests, Mann–Whitney *U* tests, Related-Samples Wilcoxon Signed Rank Tests, and unpaired *T*-tests) as appropriate. Relative Risks for complications or adverse outcomes were calculated both as Unadjusted RR and Adjusted RR after adjustment for clinically relevant confounders.

## Results

### Patient distribution

**Figure 1:**
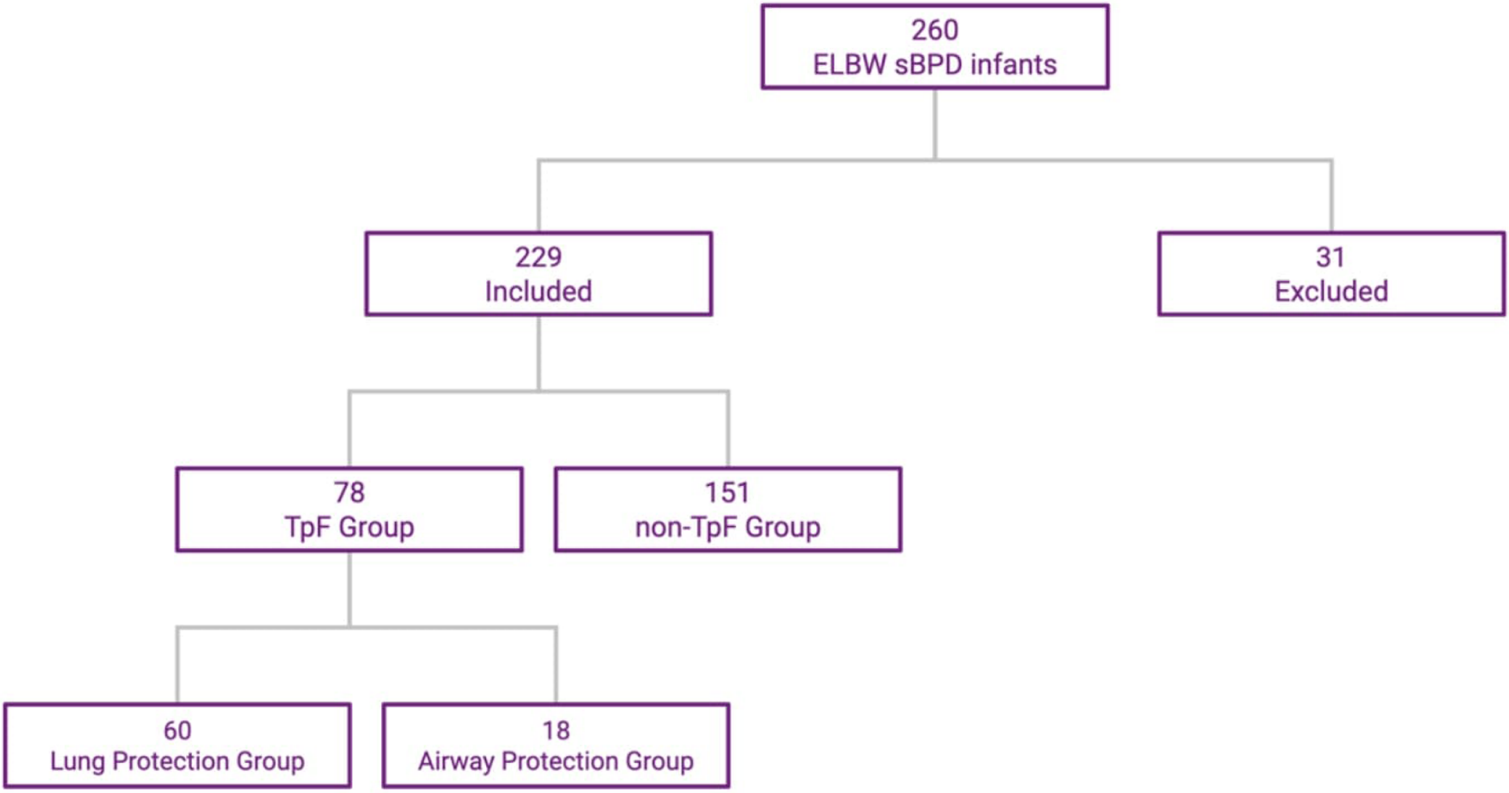
ELBW infants distribution in TpF and non-TpF groups. This flowchart shows the selection and distribution of the two cohorts.

Of the 260 ELBW infants admitted to the NICU and who survived to discharge, 31 were excluded (15 from the TpF group and 16 from the Non-TpF group) based on the exclusion criteria. Of the 229 patients included, 78 were in the TpF group, and 151 were in the non-TpF group (Figure 1).

### Patient characteristics of the 229 infants in the TpF group and the Non-TpF group

Table 1 shows the baseline comparisons of the TpF (*n* = 78) and Non-TpF groups (*n* = 151). There were no differences between the groups for sex or race. However, the TpF group had significantly lower birth weight (BW) and gestational age (GA) at birth. The morbidity characteristics were also significantly different between the two groups (Table 2). The TpF group had a considerably higher degree of illness, as manifested by higher morbidities such as Patent Ductus Arteriosus (PDA), PDA ligation, and Retinopathy of Prematurity (ROP). The TpF group needed more therapy, with a significantly higher need for inhaled nitric oxide (iNO) therapy, postnatal corticosteroids, diuretics, bronchodilator nebulizers, and anti-reflux medications (Table 2). The TpF group's RSS was also significantly higher than the non-TpF group's RSS at 36 weeks CGA (Table 2).

**Table 1:** Characteristics of ELBW in TpF group and non-TpF group.

	TpF (*n* = 78)	Non-TpF (*n* = 151)	*p*-value
GA at Birth (wk)	25.01 ± 1.58	25.77 ± 1.84	**0.003**
BW (gm)	691 ± 143	759 ± 151	**0.001**
Place of Birth			
Hartford Hospital	46 (59.0%)	128 (84.8%)	**<0.001**
Other	32 (41.0%)	23 (15.2%)	
Sex			
Male	49 (62.8%)	88 (58.3%)	0.506
Female	29 (37.2%)	63 (41.7%)	
Race			
Caucasian	26 (33.3%)	55 (36.4%)	0.248
Black	25 (32.1%)	49 (32.5%)	
Hispanic	22 (28.2%)	30 (19.9%)	
Asian	4 (5.1%)	6 (4.0%)	
Other/Unknown	1 (1.3%)	11 (7.3%)	

Data shown as Mean ± SD or N (%).

GA, gestational age; BW, birth weight.

Bold values indicate significant *p*-values.

**Table 2:** Morbidity characteristics of TpF group and non-TpF group.

	TpF (*n* = 78)	Non-TpF (*n* = 151)	*p*-value
PDA	56 (71.8%)	86 (57%)	**0.028**
PDA ligation	28 (35.9%)	27 (17.9%)	**0.002**
ROP	61 (78.2%)	81 (53.7%)	**0.001**
Anti-Reflux Meds	49 (62.8%)	60 (39.7%)	**<0.001**
Nebulizers	36 (46.2%)	15 (9.9%)	**<0.001**
Oral Diuretics	75 (96.2%)	124 (82.1%)	**0.003**
iNO therapy	11 (14.1%)	5 (3.3%)	**0.002**
Hydrocort/Pred	61 (78.2%)	65 (43%)	**<0.001**
Dexamethasone	58 (74.3%)	35 (23.2%)	**<0.001**
RSS (average)^a^	5.768	0.714	**<0.00001**
LOS (days)	143.76 ± 35.76	106.18 ± 25.56	**<0.001**
GA @ DC (wk)	46.10 ± 4.68	41.31 ± 3.24	**<0.001**

Data shown as N (%) or Mean ± SD.

PDA: patent ductus arteriosus; ROP: retinopathy of prematurity; iNO: inhaled nitric oxide; Hydrocort/Pred, hydrocortisone/prednisolone; RSS, Respiratory Severity Score (product of Mean Airway Pressure and FiO_2_/100); LOS, Length of stay; GA@DC, Gestational age at discharge.

^a^ RSS for non-TpF group at 36 weeks CGA, for TpF group at 36 weeks CGA and initiation of TpF.

Bold values indicate significant *p*-values.

After the TpF group had completed its feeding regimen, the final pre-discharge outcomes of the two groups were compared (Table 3). As expected from the pre-TpF status, the incidence of LPHN and the need for GT and TT were significantly higher in the TpF group (Table 3). However, after adjusting for the GA, BW, and pre-TpF RSS, the adjusted relative risk for GT and LPHN was no longer significant, but the need for TT remained significantly higher in the TpF group (Table 3).

**Table 3:** Outcomes for LPHN, GT, and TT in TpF group and non-TpF group.

	TPF (*n* = 78)	Non-TpF (*n* = 151)	*X*^2^ *p*-value	Unadjusted RR	Confidence Interval	Adjusted* RR	Confidence Interval
LPHN	18 (23.1%)	11 (7.3%)	0.0006	3.168	[1.575, 6.370]	1.725	[0.515, 5.775]
GT	34 (43.6%)	40 (26.5%)	0.008	1.646	[1.141, 2.374]	1.284	[0.500, 3.299]
TT	14 (17.9%)	2 (1.3%)	0.000002	13.551	[3.159, 58.128]	12.044	[2.092, 69.354]

*X*^2^ *p*-value, *p*-value from chi-squared analysis; Unadjusted RR, unadjusted relative risk; Adjusted RR, adjusted relative risk.

* Adjusted for GA, BW, and pre-TpF RSS scores.

### Sub-group analysis of the 78 infants in the TpF group

#### Indication for TpF feeds and outcomes

**Figure 2:**
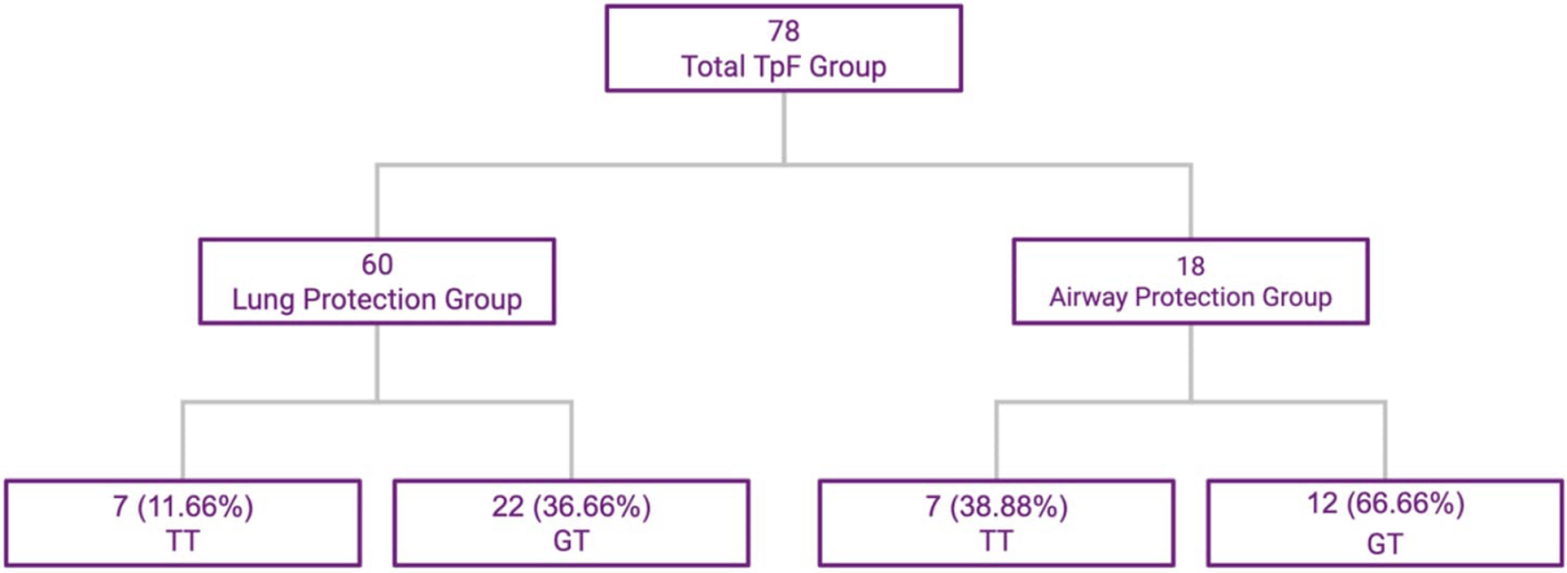
TpF patient subgroups based on indication, and outcomes of TT and GT. This flowchart shows the subdivisions within the study group.

The 78 infants in the TpF group were further evaluated based on primary indication for TpF. In a large majority of infants in this group, the indication for starting TpF was to protect their lungs from continuing injury based on clinical findings of worsening respiratory distress, tachypnea, or pulmonary edema probably related to GER with microaspiration and lung inflammation (*Lung Protection Group—60/78*). The primary indication in the other 18 infants were airway issues (*Airway Protection Group—18/78*) after demonstrable aspiration or airway compromise, with diagnoses such as subglottic stenosis, vocal cord paresis or palsy on bronchoscopy or frank aspiration noted on the modified barium swallow test (MBS) (Figure 2). There was some overlap between the 2 groups with minor penetration or suspected aspiration included in the Lung Protection Group whereas demonstrable aspiration on MBS was included in the airway protection group.

The infants' pre-discharge outcomes were significantly associated with the primary indication for TpF (Table 4). Infants in the Airway Protection Group had a significantly higher risk for GT and TT. Even after adjusting for GA, BW, and pre-TpF RSS, the adjusted relative risk for GT and TT remained significant (Table 4). There was no difference in the incidence of LPHN within the two groups (Table 4).

**Table 4:** Outcomes for LPHN, GT, and TT for subgroups within TpF group (lung protection and airway protection).

	Lung protection group (*n* = 60)	Airway protection group (*n* = 18)	*X*^2^ *p*-value	Unadjusted RR	Confidence Interval	Adjusted RR*	Confidence Interval
LPHN	11/60 (18.33%)	7/18 (38.88%)	0.069	0.471	[0.214, 1.036]	0.208	[0.034, 1.263]
GT	22/60 (36.66%)	12/18 (66.66%)	0.024	0.550	[0.345, 0.877]	0.214	[0.049, 0.934]
TT	7/60 (11.66%)	7/18 (38.88%)	0.008	0.300	[0.121, 0.742]	0.129	[0.028, 0.598]

*X*^2^ *p*-value, *p*-value from chi-squared analysis; Unadjusted RR, unadjusted relative risk; Adjusted RR, adjusted relative risk.

* Adjusted for GA, BW, and pre-TpF RSS scores.

Surgical interventions for feeding included, GT alone, or with fundoplication or gastro-jejunostomy placement as clinically indicated.

#### Timing of respiratory response after TpF initiation

**Figure 3:**
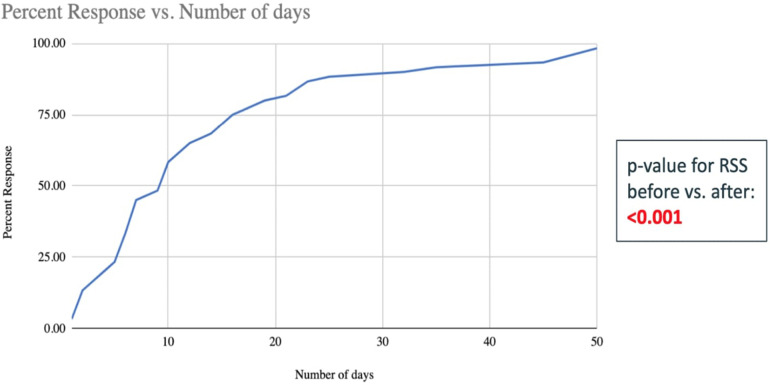
Number of days taken to improve the RSS scores of the 60 infants in the TpF group who had transpyloric feeds started for lung protection (shown in percent infants cumulative). The x-axis is the number of days of TpF, and the y-axis shows the percent of infants with sBPD whose RSS improved after TpF (the percentage is cumulative). This graph demonstrates that at approximately 20 days, more than 75% of patients have decreased their RSS after being started on TpF.

The need for respiratory support (based on RSS pre- and post-treatment) decreased significantly in the TpF group compared to the non-TpF group (*p* < 0.001) (Figure 3). To evaluate the timing of respiratory improvement after TpF, we only evaluated the Lung Protection group (60 infants) because the Airway Protection Group did not necessarily have a poor baseline respiratory status. In the Lung Protection Group, there was a significant decrease in respiratory support with improvement of symptoms (tachypnea and obstructive apnea) within 45 days (median 9 days, range 1–45 days) in 57 out of 60 infants (95%).

Within the Lung Protection Group (*n* = 60), the quickest response (improvement in <3 days after initiation of TpF) was seen in those infants for whom the indication was severe obstructive apnea. Significant improvement within 7 days of initiation of TpF was seen in 27 out of 60 infants, among whom 12 (45%) had sBPD with tachypnea as their predominant symptom, and 15 (55%) had suspected GER as their primary issue (Figure 3).

The primary goal of TpF in the Airway Protection Group was to support them while awaiting TT or GT or transfer to PICU. The goal in this group was not to wean support.

In infants who did not need surgical intervention, after the trial of TpF was completed the “J” tube was pulled back to the stomach and continuous feeding through the stomach was resumed. We did not encounter any problems with tolerance after the position of the feeding tube was changed to the stomach.

#### The clinical need for initiation of TpF earlier than the proposed guideline

In evaluating the timing and indication for TpF, we found that although the mean CGA for starting TpF was 36 weeks ±5 days, the range was from 28 4/7 weeks to 42 weeks. Of the 10 infants that were in the TpF group at <36 weeks CGA, the earlier need for TpF was necessitated by severe symptoms of GER with obstructive spells or aspiration pneumonia in 6 (60%) infants and rapidly increasing severity of lung disease alone for 4 (40%) infants.

#### Complications related to TpF

We did not encounter any gastrointestinal complications, such as NEC, perforation, or hypertrophic pyloric stenosis, in TpF patients. There was no need to decrease the caloric density of feeds and optimal nutrition could be provided to all infants irrespective of TpF use. The use of TpF did not adversely influence the growth and nutrition of infants.

## Discussion

Even with institutional guidelines in place for the use of TpF in ELBW infants at around 36 weeks CGA, our retrospective cohort study found that TpF was initiated between 28 4/7 and 42 weeks CGA and was used more selectively in the sicker and smaller infants with a higher morbidity load (as determined by their higher RSS, higher incidence of PDA, PDA ligation, ROP and greater need for medications such as steroids and diuretics—Table 2). Therefore, when the TpF and the non-TpF groups were compared, it was not surprising that the TpF group was associated with significantly higher morbidity as determined by the higher incidence of LPHN and greater need for surgical intervention for GT or TT placement.

A recent retrospective multicenter study showed that TpF is associated with adverse in-hospital outcomes such as increased TT, prolonged hospital stay, and death in infants with sBPD. These infants were studied at specific time points at 36, 44, and 50 weeks, and the duration and indications for TpF varied greatly from center to center (10). Our patients also had a higher incidence of TT, LPHN, GT, and longer durations of hospital stay, but they were sicker than the non-TpF group at the start of the study. They were also on TpF for specific indications and duration, and we could follow at what point in time they had a response and improvement in their respiratory status. Moreover, in our study, we could document the RSS in both the non-TpF and TpF groups at 36 weeks or before initiation of TpF.

An important observation made in our study was that the infants started on TpF had a significant improvement in respiratory status within a few days of initiation of this mode of feeding. The effects were noted early, within 5–7 days, especially in infants with demonstrable GER-associated obstructive apnea. Even in infants with chronic respiratory issues unassociated with demonstrable GER, a slower but sustained improvement was noted by a median of 14 days of TpF (Figure 3).

The role of proven aspiration in the pathogenesis of sBPD has been under evaluation for many decades. Radford et al. showed that GER, as diagnosed by pH probe studies and lipid-laden macrophages in tracheal secretions, was a factor in developing severe lung disease in a select small sample of 12 infants (23). Anti-reflux surgery in infants with BPD was associated with a statistically significant reduction in median respiratory support and FiO_2_ shortly after surgery, even in infants who had developed sBPD (24). Hrabovsky et al. found that infants with sBPD with GER and/or aspiration had clinical improvement after medical (smaller feeds and upright positioning) or surgical (fundoplication) treatment (25). GER-related obstructive apneic events have been shown to respond to the initiation of TpF within 72 h, as demonstrated in a prospective study by Biswas et al. (26).

Farhath et al. first suggested the role of 'silent' aspiration or microaspiration in the evolution of sBPD in a series of two clinical studies. Their first study demonstrated the presence of pepsin in serial tracheal samples in most ventilator-dependent premature infants on enteral feeds (27). In their second study, they correlated tracheal pepsin concentration with the severity of BPD (4, 27). Thus, prevention of ongoing ’silent' or demonstrable pulmonary aspiration of gastric contents may help decrease the burden of lung injury and help manage sBPD. Our findings of an immediate and consistent improvement in RSS after initiating TpF could thus be explained by a potential decrease in micro aspiration.

There have been competing findings of improved or worsening hypoxemia with the use of TpF. In a small single-center N-of-1 trial of 15 infants, Jensen et al. found that TpF caused more hypoxemia than gastric feeds. Interestingly, two of the infants could not complete the study because they had worse hypoxemia with gastric feeds (20). Srivatsa et al. reported contrary findings in a retrospective study of 56 infants whose oxygen requirements were evaluated for the 96 h prior to and after the initiation of TpF. They reported a significant improvement in oxygenation with TpF, especially in the non-intubated infants (17). Our study's findings were consistent with those of Srivatsa et al. We found that 97% of infants in our study had improved RSS after initiation of TpF, and the improvement started as early as 72 h.

The reason for starting TpF therapy is also essential in evaluating its efficacy. As shown in our study, two main categories of conditions prompted TpF. Infants with demonstrable problems with aspiration due to abnormality or dysfunction of the upper airway (*Airway Protection Group*) and in those to decrease lung injury due to presumed microaspiration (*Lung Protection Group*). The goal of the *Airway Protection Group* was to bridge the time until a surgical procedure could be done, such as a GT or TT (Figure 2). Therefore, it is not surprising that the incidence of TT and GT placement was significantly higher in the *Airway Protection Group*. In the *Lung Protection Group*, we have shown a significant improvement in respiratory status, with improvement in the RSS score and a decrease in other respiratory morbidities. In evaluating studies of TpF efficacy, we suggest that the reason for TpF initiation be critically assessed.

Reported complications of TpF include the development of hypertrophic pyloric stenosis (28), intestinal perforation and feeding intolerance. However, in our limited study of 78 infants, no such complications were found.

Very few randomized controlled trials have compared TpF vs. gastric feeds in ELBW infants. A meta-analysis found that reported data do not provide sufficient evidence of the beneficial effect of TpF for preterm infants, while some evidence of harm exists, including a higher risk of gastrointestinal disturbance and mortality. However, the authors agree that there were methodological weaknesses in the included trials and that the results should be interpreted cautiously (16).

Although TpF was more frequently used in the sicker ELBW infants, RSS significantly improved after its initiation. TpF could play an essential role in the management of a subset of ELBW infants with sBPD with GER-associated obstructive apnea and/or microaspiration. When used, infants with TpF show a significant improvement in their respiratory status by 14 days of initiation, and 80% of responders could be identified by 21 days after initiation (Figure 3). This information could help determine the optimal duration, if a trial of TpF therapy were to be considered for an individual infant. Prospective studies are needed to understand better if a subset of patients would benefit the most from TpF, what the minimal duration for a TpF trial should be, and what impact TpF would have on these infants' ultimate nutrition and growth. The safety of this therapy also needs to be carefully evaluated.

A significant limitation of our study is that it is a retrospective, single-center study, and the duration of TpF varied based on attending preference. Despite the use of institutional guidelines, we have shown a selection bias with the sicker infants receiving TpF. It appears that in several infants, TpF was used to manage the sickest infants when all other therapy failed. We still feel that these results may be reproducible in similar level 4 institutions who manage ELBW infants with sBPD. It may well be that the use of TpF around 36 weeks CGA in the course of evolving lung disease in ELBW infants may be too late to prevent lung injury. Clinical trials focused on earlier initiation of TpF in at-risk infants may be worth conducting as a strategy to avert sBPD.

## Data Availability

The raw data supporting the conclusions of this article will be made available by the authors, without undue reservation.
